# CNOT7 depletion reverses natural killer cell resistance by modulating the tumor immune microenvironment of hepatocellular carcinoma

**DOI:** 10.1002/2211-5463.12836

**Published:** 2020-04-02

**Authors:** Chongren Ren, Xiaojing Ren, Dujuan Cao, Haichao Zhao, Zhensheng Zhai, Huiyu Li, Yanjun Li, Xifeng Fu, Jiefeng He, Haoliang Zhao

**Affiliations:** ^1^ Shanxi Bethune Hospital TaiYuan China; ^2^ Graduate College of Shanxi Medical University TaiYuan China

**Keywords:** CNOT7, hepatocellular carcinoma, HepG2, STAT1, TGF‐β1

## Abstract

A major obstacle to effective cancer immunotherapy is the tumor immune microenvironment. Natural killer (NK) cell resistance has been suggested as a primary cause of poor prognosis in hepatocellular carcinoma (HCC), which seemingly correlates with CNOT7 overexpression. CNOT7, a cytoplasmic mRNA deadenylase that is highly expressed in HCC, may regulate cytokine transforming growth factor‐β1 (TGF‐β1) secretion by controlling nuclear factor‐κB subunit p65 trafficking. CNOT7 depletion suppresses TGF‐β1 secretion in HCC and promotes interferon‐γ (IFN‐γ) secretion by NK cells, and we previously demonstrated that CNOT7 depletion reversed IFN‐γ resistance in HCC cells. Therefore, we hypothesized that CNOT7 depletion might reverse NK cell resistance by influencing the tumor immune microenvironment of HCC. To test this hypothesis, we examined the correlation between CNOT7, STAT1, TGF‐β1 and IFN‐γ expression with hepatitis B virus‐related cirrhosis and HCC with hepatitis B virus‐related cirrhosis. We found that modulation of CNOT7 expression alters TGF‐β1 secretion in HCC and IFN‐γ secretion in NK cells. We also examined the effects of NK cells in HepG2 cells with *CNOT7* knockdown, which showed that NK cell surface CD107a expression is up‐regulated and caspase‐3 expression is significantly enhanced in CNOT7‐deficient HepG2 cells. Overall, our results show that knockdown of *CNOT7* expression reverses NK cell resistance in HCC cells. Therefore, CNOT7 depletion has potential as a new adjuvant therapy in immunotherapy for HCC.

## Graphical abstract

Natural killer (NK) cell resistance is a primary cause of poor prognosis in hepatocellular carcinoma (HCC), which correlates with CNOT7 overexpression. Tumor‐derived transforming growth factor‐β1 created a strong inhibitory network and impaired NK cells. CNOT7 depletion could suppress transforming growth factor‐β1 secretion by controlling nuclear factor‐κB subunit p65 trafficking in HCC. We found that CNOT7 depletion may reverse NK cell resistance in HCC cells.

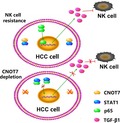

**Abbreviations**
7‐AAD: 7‐aminoactinomycin D
CFSE: carboxyfluorescein succinimidyl amino ester
co‐IP: coimmunoprecipitation
CON: healthy controls
E:T: effector‐to‐target
HBC: HBV‐related cirrhosis
HBV: hepatitis B virus
HCC: hepatocellular carcinoma
HCCBC: hepatocellular carcinoma with HBV‐related cirrhosis
NC: negative control
NF‐κB: nuclear factor‐κB
SD: standard deviation
TCGA: The Cancer Genome Atlas
TGF‐β1: transforming growth factor‐β1

Hepatocellular carcinoma (HCC) is the fourth deadliest neoplastic disease in the world [1]. HCC originates from hepatic cirrhosis, which is primarily ascribed to chronic infections, such as hepatitis B or C virus infections, followed by other etiological causes of cirrhosis, such as alcohol abuse and fatty liver disease associated with metabolic defects [2, 3]. Chronic inflammation of the liver is the principal cause of HCC development by intensifying immunosuppression and angiogenesis, which results in a kind of microenvironment for tumor cell growth and invasion [4]. Once a tumor forms, the interaction of tumor cells and the host immune system establishes an active intrinsic immunosuppression microenvironment that fuels tumor growth. This microenvironment is one of the main hurdles for successful cancer immunotherapy [4, 5].

Natural killer (NK) cells congregate in the human liver; the number of NK cells in the liver is roughly two to five times greater than in peripheral blood [6]. In addition, compared with NK cells in peripheral blood, NK cells in a healthy liver have distinct phenotypic features and functional capabilities, containing significantly higher cytotoxic activity against tumor cells [7, 8, 9]. Tumor‐associated NK cells in patients with HCC exhibit phenotypic changes, including the absence of interferon‐γ (IFN‐γ) production and defects in degranulation capabilities, as well as cytokine secretion and cytotoxicity [10]. Further, immunomodulatory factors in the tumor microenvironment, such as transforming growth factor‐β1 (TGF‐β1), contribute to the inhibited expression of stimulatory receptors, promote expression of inhibitory receptors and impair secretion of cytotoxic proteins, which impair NK cell‐mediated antineoplastic activity and reinforce tumor immune evasion [11, 12, 13]. Therefore, appropriate antineoplastic immunotherapy should ameliorate the depression microenvironment and enhance NK cell functions.

CNOT7 is a key cytoplasmic mRNA deadenylase in metazoans [14, 15]. Our previous study revealed that CNOT7 mRNA and protein expressions were remarkably up‐regulated in HCC tissues compared with healthy liver tissues. CNOT7 depletion promoted STAT1 expression and inhibited STAT3 expression in HCC [16]. The susceptibility of HCC cells to NK cell cytolysis is improved by inhibition of *STAT3* expression [17]. Even more important, mice vaccinated with STAT3‐blocked HCC cells effectively damage tumor‐induced immunosuppression, leading to a valid antitumor effect [17, 18].

To discover the role of CNOT7 in NK cell resistance of HCC, we measured plasma levels of TGF‐β1 and IFN‐γ in patients with HBV‐related cirrhosis (HBC) and patients with HCC with HBV‐related cirrhosis (HCCBC). In addition, IFN‐γ and TGF‐β1 concentrations were determined in the tumor, adjacent nontumor and healthy liver tissues of patients with HCCBC. CNOT7 and STAT1 expression levels were determined in tumor and healthy tissues of patients with HCCBC. Further, the viability of four HCC cell lines was examined after coculture with NK cells. The expression levels of CNOT7 and STAT1, and TGF‐β1 concentrations were evaluated in four HCC cell lines and compared with the human liver cell line, L02. Furthermore, we knocked down *CNOT7* expression in HepG2 cells (HepG2^shCNOT7^), and we compared the effects of NK cells on HepG2^shCNOT7^ and HepG2 cells, and measured TGF‐β1 and IFN‐γ levels in coculture supernatants. We expected our findings to deepen our understanding of the contribution of CNOT7 to NK resistance in HCC and to reveal new potential targets for HCC treatment.

## Materials and methods

### Study subjects

The study subjects, including 58 patients pathologically diagnosed with HCCBC, 60 patients with HBC and 60 healthy controls (CON), were enrolled at the Shanxi Bethune Hospital between September 2013 and January 2019. Patients were excluded based on the following criteria: age <30 years or >65 years; hepatic function Child–Pugh class C; any immunotherapy during the 6 months before sampling; hepatitic C virus, hepatitis D virus or HIV infection; or drug abuse or autoimmune hepatitis. Patients with HCCBC with secondary tumors, lymph node involvement, multiple tumors, metastasis or incomplete resection were also excluded. All subjects were diagnosed according to the international diagnostic criteria by clinical, radiological and histological diagnosis. The study was authorized by the ethics committee of the Shanxi Medical University, and the study methodologies conformed to the standards set by the Declaration of Helsinki. After being notified of the contingent risks of the study, each subject signed an informed consent. The clinical characteristics of eligible subjects are shown in Table 1. Fresh tumor specimens were drawn from the 58 subjects with HCCBC. Of these, 49 paired specimens of cirrhotic hepatic tissue (distal to the tumor site 5 cm), adjacent cirrhotic hepatic tissue (distal to the tumor site 1 cm) and tumor tissues were from the same subjects.

**Table 1:** Clinical characteristics of eligible subjects. AFP, α‐fetoprotein.

	HCCBC (*n* = 58)	HBC (*n* = 60)	CON (*n* = 60)
Age (≥50/<50 years)	36/22	35/25	35/25
Sex (male/female)	41/17	40/20	40/20
HBV–DNA median level (log_10_ copies per mL)	4.73 ± 2.64^a^	5.63 ± 6.27^a^	—
AFP (≥400/<400 µg·L^−1^)	47/11	7/53	—
Tumor diameter (≥5/<5 cm)	37/21	—	—
Differentiation (high‐moderate/low)	42/16	—	—

^a^ Non‐normal distribution, data are expressed as the median ± quartile range.

### Survival analysis of *CNOT7* genes in the TCGA data

We performed survival analysis for *CNOT7* genes using The Cancer Genome Atlas (TCGA) data (http://ualcan.path.uab.edu/index.html), screening 273 samples of HCC with *CNOT7* low/medium expression and 92 HCC samples with *CNOT7* higher expression [19]. 

### Preparation of tumor, adjacent and normal tissue culture supernatants

After removing the fat and blood clots from the freshly collected tumor, adjacent nontumor and healthy liver tissues, specimens were weighed and washed with ice‐cold Dulbecco’s modified Eagle’s medium (DMEM; Boster Biological Technology, Wuhan, China) solution three times. The tissues were cut into small chunks on dry ice using a surgical scalpel, ground and placed into 24‐well culture plates. A solution of DMEM containing 1% penicillin and streptomycin (Solarbio Technology, Beijing, China) was added to each well. The culture plates were incubated in the presence of 5% CO_2_ at 37 °C for 24 h. Cells and debris were discarded by centrifugation. Culture supernatants were collected.

### Cell lines and cell culture

NK‐92MI cells (Chinese Academy of Sciences, Kunming, China) were grown in Minimum Essential Medium alpha medium (donated by Kunming Cell Bank) according to the accompanying guidelines. HepG2, SMMC7721, Huh7, PLC/PRF/5 and L02 cells (Chinese Academy of Sciences, Shanghai, China) were routinely grown in DMEM added with 10% FBS (Sijiqing Biological Engineering Materials, Hangzhou, China) and 1% penicillin–streptomycin (Solarbio) at 37 °C in the presence of 5% CO_2_.

### Cytokine assay and immunohistochemical analysis

Plasma and tissue levels of IFN‐γ and TGF‐β1 were detected using ELISA kits (Boster Biological Technology). The sensitivity of the ELISA kits for IFN‐γ and TGF‐β1 was 7 pg·mL^−1^. CNOT7 and STAT1 expression levels in tumor and normal liver tissues were analyzed by immunohistochemistry. The paraffin‐embedded tissue sections (4 μm) were incubated with primary antibodies against CNOT7 (diluted 1 : 200; Santa Cruz Biotechnology, Santa Cruz, CA,USA) and STAT1 (diluted 1 : 200; Cell Signaling Technology, Beverly, MA, USA). The secondary antibodies were detected with biotinylated goat anti‐(mouse/rabbit IgG) serum and visualized using the streptavidin–biotin complex method (SABC and DAB Histostain Kits; Boster Biological Technology). We used a specimen that was stained consistently as positive control. Two pathologists scored the staining intensity in a blinded manner: 0 = no staining; 1 = weak, complete cytoplasm staining in < 10% of cells; 2 = medium, complete cytoplasm staining in ≥10% of cells, or intense complete cytoplasm staining in ≤ 30% of cells; and 3 = strong, intense uniform cytoplasm staining in >30% of cells. Scores of 0 and 1 were regarded as ‘low’ expression, and scores of 2 and 3 were regarded as ‘high’ expression [20].

### Transfection of HepG2 cells

HepG2 cells (2 × 10^5^ per well) were cultured in a six‐well plate for 24 h before transfection with CNOT7 shRNAs to knock down *CNOT7* expression, using Lipofectamine 2000 (Invitrogen, Grand Island, NY, USA), following the manufacturer’s directions. Four shRNAs were encoded by plasmids containing the puromycin‐resistant gene specifically targeting *CNOT7* (shCNOT7 #1, 5′‐TACTAACAACATCTGGTAT‐3′; #2, 5′‐TGACTATCAATACCAACTA‐3′; #3, 5′‐TGACTATCAATACCAACTA‐3′; and #4, 5′‐GTGTAATGTAGACTTGTTA‐3′), and nonspecific shRNAs as a control (5′‐TTCTCCGAACGTGTCACGT‐3′) were purchased from GeneChem (Shanghai, China). The knockdown efficacy of the shRNAs was analyzed by quantitative real‐time PCR and western blot analysis. After the 48‐h transfection, selection of stably transfected cells was initiated in medium containing 450 ng·mL^−1^ puromycin (Solarbio).

### Treatment of NK cells with supernatant from tumor cells

After the establishment of stably transfected HCC cells for 24 h, the puromycin‐containing medium was discarded. HCC cells were washed with PBS. The HCC cells were then cultured in fresh Minimum Essential Medium alpha medium for an additional 24 h. Cells and debris were discarded by centrifugation, and supernatants were collected. NK‐92MI cells were cultured in the supernatant for 12 h at a supernatant‐to‐medium ratio of 1 : 1 (v/v). The culture solution was collected and centrifuged at 15 000 ***g***.

### Cell proliferation assay

The effect of NK‐92MI cells on cell proliferation in the PLC/PRF/5, Huh7, SMMC7721 HepG2, HepG2^ns^ (nonspecific shRNA) and HepG2^shCNOT7^ cell lines was evaluated by MTT assay (Sigma‐Aldrich, St. Louis, MO, USA). Cells (4 × 10^3^ per well) were cultured in 96‐well plates overnight. The cells were serum starved for 12 h. NK‐92MI cells were added into the plates at various effector‐to‐target (E:T) ratios for 12 h. The cells were washed to remove the medium containing NK‐92MI cells and debris, and were then supplied with serum‐free culture medium. Ten microliters of MTT reagent was added into each well, and the cells were incubated for 4 h. Then the medium was discarded, 100 µL DMSO was added into each well and the plates were incubated for another 1 h. The absorbance at 490 nm was determined using a Victor X5 Multilabel Reader spectrophotometer, and data were obtained from five replicate wells for each cell line.

### Cell cytotoxicity assay

NK‐92MI cell cytotoxicity toward HepG2 cells was detected in carboxyfluorescein succinimidyl amino ester (CFSE)/7‐aminoactinomycin D (7‐AAD) by flow cytometry. After labeling with CFSE (KeyGen BioTECH, Nanjing, China) for 15 min at 37 °C, the HCC cells (4 × 10^5^ per well) were washed with medium and seeded in six‐well plates for adherent culture. NK‐92MI cells were added at various E:T ratios. HCC cells incubated without NK cells were included to measure basal cell death. After 4 h, the cells were collected, washed with PBS and incubated with 7‐AAD (KeyGen BioTECH) for 15 min at ambient temperature in the dark. Then the cells were analyzed by flow cytometry.

### Detection of NK cell cytotoxicity with CD107a

NK‐92MI cells were incubated with target cells at a ratio of 10 : 1 along with a negative control (NC), which consisted of NK cells without coculture with target cells. After 2 h, monensin (2 μm) was added to the culture plate for 1.5 h. Then NK‐92MI cells were extracted and purified from culture medium with discontinuous density gradient centrifugation, and NK‐92MI cells were labeled with phycoerythrin–Cy5–CD107a and FITC–CD56 (both from Cell Signaling Technology) for 30 min at 37 °C and detected by flow cytometry. We used 2.5 μg·mL^−1^ phorbol myristate acetate and 0.5 μg·mL^−1^ ionomycin as positive controls.

### Quantitative real‐time PCR analysis

Total RNA was isolated using SuperEnhanced TRIzol reagent (Sangon Biotech, Shanghai, China) and reverse transcribed into cDNA using a Fermentas K1622 RevertAid First Strand cDNA Synthesis Kit (Thermo Scientific, Rockford, IL, USA). PCRs were run using FastStart Universal SYBR Green Master (ROX) (Roche, Mannheim, German) on an ABI StepOnePlus qPCR system (Applied Biosystems, Foster, CA, USA). Each sample was tested in triplicate. The relative fold change in gene expression was calculated by the $2^{-\Delta \Delta C_{t}}$ method. Primer sequences were as follows: *GAPDH*, forward 5′‐CTCTGCTCCTCCTGTTCGAC‐3′ and reverse 5′‐TTAAAAGCAGCCCTGGTGAC‐3′ (104 bp); *CNOT7*, forward 5′‐GAGGAAGCCAACAAGCAGTC‐3′ and reverse 5′‐GTTCGAGGGATTCAACCAGA‐3 (105 bp); *STAT1*, forward 5′‐CCGTTTTCATGACCTCCTGT‐3′ and reverse 5′‐TGAATATTCCCCGACTGAGC‐3 (228 bp); *caspase‐3*, forward 5′‐CTGCCGGAGTCTGACTGGAA‐3′ and reverse 5′‐ATCAGTCCCACTGTCTGTCTCAATG‐3′ (97 bp); *TGF‐β1*, forward 5′‐GGGGTACCCCAGATGGAGAGAGGACTG‐3′ and reverse 5′‐CCCAAGCTTGGGCAGTCTTGGCTGGGTGCG‐3′ (103 bp); *NF‐κb p65*, forward 5′‐GGCCATGGACGAACTGTTCCC‐3′ and reverse 5′‐GGAGGGTCCTTGGTGACCAG‐3′ (256 bp).

### Western blotting and coimmunoprecipitation

Cytosolic and nuclear proteins were extracted by Nuclear‐Cytosol Extraction Kit (Beyotime Institute of Biotechnology, Shanghai, China). Protein concentrations were detected by the bicinchoninic acid method. Proteins (20–40 μg) were separated by 8–15% SDS/PAGE and transferred onto polyvinylidene fluoride membranes (Millipore, Billerica, MA, USA). The membranes were washed with Tris‐buffered saline with Tween (TBST) and incubated with blocking buffer (TBST containing 50 g·L^−1^ skim milk) at 37 °C for 3 h. The membranes were incubated with primary antibody against CNOT7 (mouse monoclonal antibody; diluted 1 : 500; Santa Cruz Biotechnology), STAT1 and nuclear factor‐κB (NF‐κB) p65 (rabbit monoclonal antibodies; diluted 1 : 1000; Cell Signaling Technology), caspase‐3 and cleaved caspase‐3 (rabbit monoclonal antibodies; diluted 1 : 1000; Abcam, Cambridge, MA, USA), and GAPDH (rabbit polyclonal antibody; diluted 1 : 5000; Bioworld Technology, Nanjing, China) at 4 °C overnight. Then the membranes were washed in TBST and incubated with horseradish peroxidase‐linked secondary antibodies (anti‐rabbit or anti‐mouse IgG; diluted 2 : 5000; Boster Biological Technology) at 37 °C for 2 h. After washing the membranes with TBST, the signals were detected using an ECL detection kit. Target protein levels relative to that of the control GAPDH were determined using the AlphaView‐FluorChemQ Gel Imaging System (ProteinSimple, Silicon Valley, CA, USA). For coimmunoprecipitation (co‐IP), 800 mg lysate was incubated with the appropriate antibody (1–2 mg) at 4 °C for 3–4 h and then incubated with Protein A/G PLUS‐Agarose beads for 1 h (Santa Cruz Biotechnology). The immunoprecipitates were washed at least three times in radioimmunoprecipitation assay lysis buffer, and the immunoprecipitates were separated by SDS/PAGE and immunoblotted with the indicated antibodies. Rabbit IgG (Santa Cruz Biotechnology) was used as an NC.

### Statistical analysis

Clinicopathological variables and CNOT7 and STAT1 expression levels were assessed by the chi‐square test. All other data were evaluated using Student’s *t*‐test or one‐way ANOVA in spss 22.0 (SPSS Statistics Software 22.0, IBM, Armonk, NY, USA) and expressed as the mean ± standard deviation (SD). The significance level was set to 0.05.

## Results

### Correlations of CNOT7, STAT1, TGF‐β1 and IFN‐γ levels with HCCBC and HBC

**Fig. 1:**
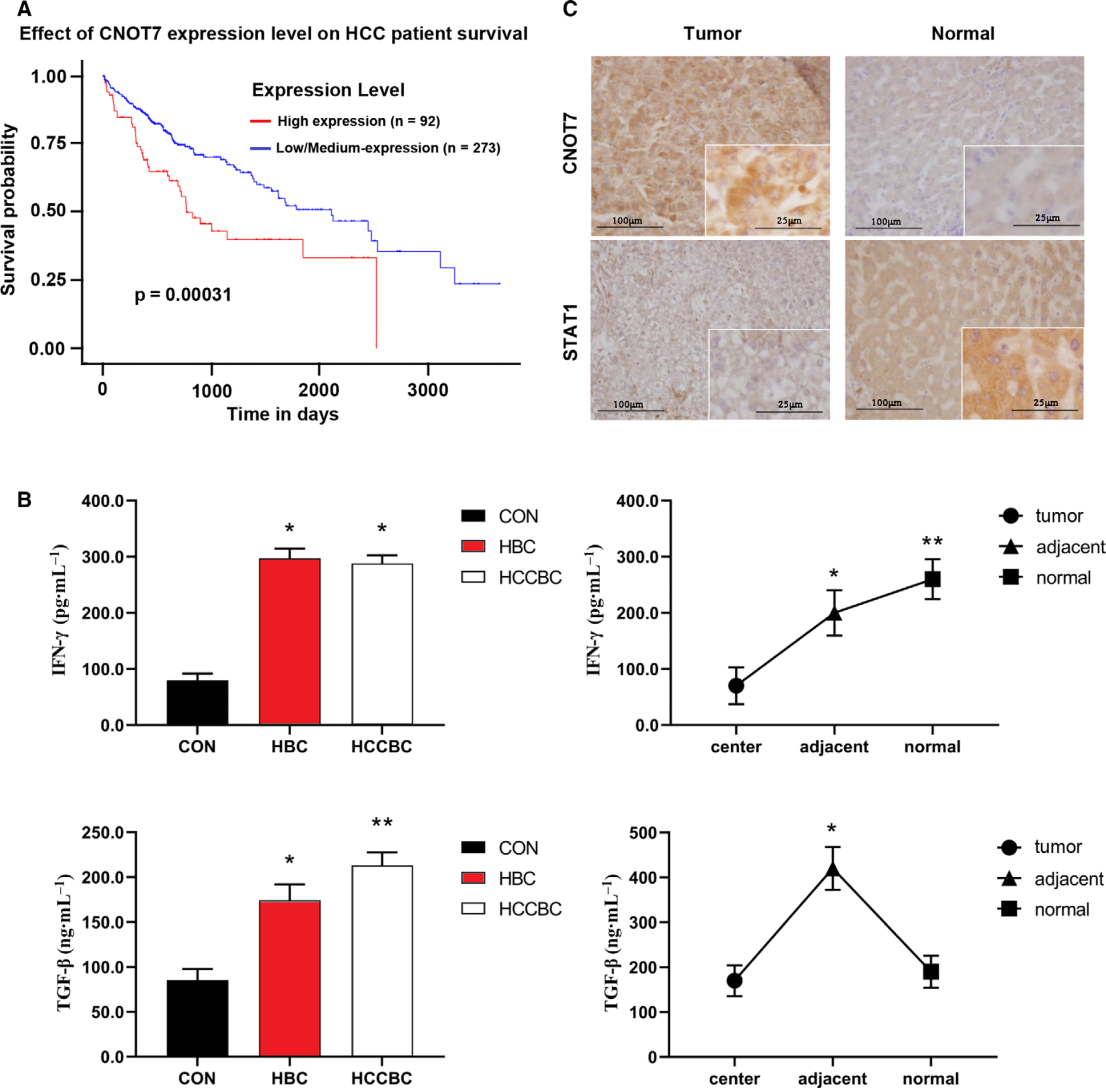
Changes in CNOT7, STAT1, TGF‐β1 and IFN‐γ levels in patients with HCCBC. (A) Correlation of CNOT7 expression level on HCC patient survival. Red lines represent the high expression of *CNOT7*, and blue lines represent low expression. (B) Quantitative data (mean ± SD) of ELISAs were shown. Multiple comparisons were made by the Student‐Newman‐Keuls*q* (SNK‐*q*) method. *P < 0.05, one‐way ANOVA. Adjacent: adjacent liver tissue; normal: healthy liver tissue; tumor: tumor tissue. (C) Immunohistochemical analysis of CNOT7 and STAT1 expression in HCCBC and cirrhotic liver tissues [original magnification, ×100 and ×400 (insets)]. Scale bars: 100 µm; 25 µm (insets). Cells with yellow‐brown staining are immune positive. Normal: cirrhotic liver tissue; tumor: tumor tissue. Values with different numbers of asterisks differ significantly (*P* < 0.05)

Statistical analysis of data downloaded from TCGA database revealed that high *CNOT7* expression is associated with poor prognosis in patients with HCC (*P* < 0.05; Fig. 1A). Plasma concentrations of IFN‐γ and TGF‐β1 were detected by ELISA in 60 CON, 60 HBC and 58 HCCBC cases. IFN‐γ concentration did not vary obviously between subjects with HBC and those with HCCBC but was higher in subjects with HBC and HCCBC than in CON. TGF‐β1 concentration was significantly higher in subjects with HCCBC than in those with HBC and CON. Tissue concentrations of IFN‐γ and TGF‐β1 were determined in 49 patients with HCCBC. TGF‐β1 concentrations were significantly higher in adjacent tumor tissues than in tumor and cirrhotic hepatic tissues, whereas IFN‐γ concentration was significantly higher in cirrhotic hepatic tissues than in tumor and adjacent tissues (*P* < 0.05; Fig. 1B). Immunohistochemistry for CNOT7 and STAT1 protein expression in 49 paired HCCBC and cirrhotic hepatic tissues (Fig. 1C) revealed that these proteins were expressed predominantly in the cytoplasm in tumor and cirrhotic hepatic tissues. CNOT7 expression was significantly higher in tumors than in cirrhotic hepatic tissues, whereas STAT1 was obviously down‐regulated in tumor tissues (both *P* < 0.01; Table 2).

**Table 2:** CNOT7 and STAT1 expression levels in tumor and normal liver tissues. High, high expression; low, low expression; normal, healthy liver tissues; tumor, tumor tissues.

	*N*	CNOT7		*P* ^a^	STAT1		*P* ^a^
		High	Low		High	Low	
Tumor	49	29	20	0.0084	15	34	0.0024
Normal	49	16	33		30	19	

^a^ Based on chi‐square test.

### NK cell resistance is the highest in HepG2 cells among HCC cell lines

**Fig. 2:**
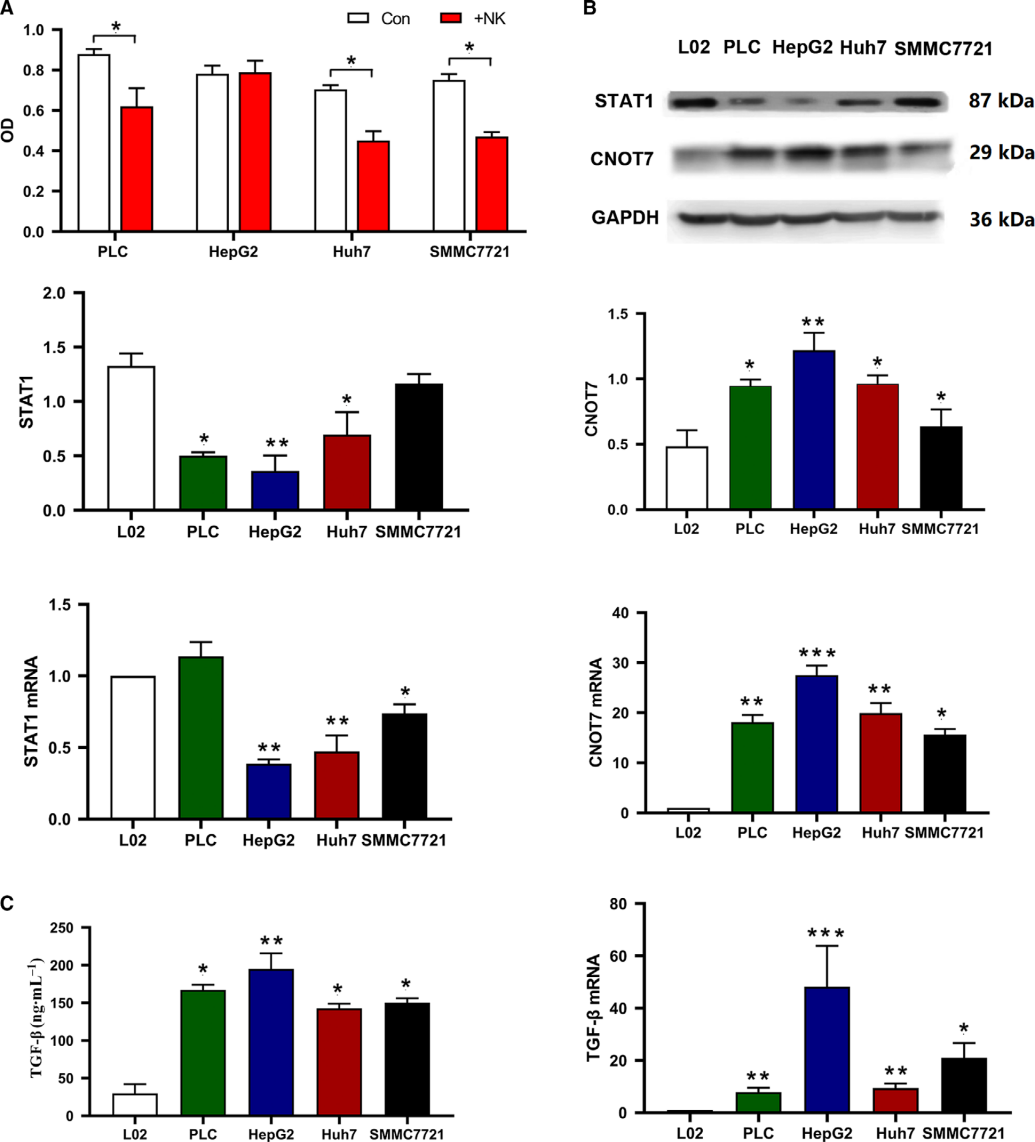
Resistance of HCC cell lines to NK cells. (A) Four HCC cell lines were cocultured with or without NK‐92MI cells at 1 : 10 ratio for 12 h. MTT assay was applied for cell proliferation detection. Quantitative data (mean ± SD) of individual groups of cells were shown. Experiments were conducted in triplicate; *P < 0.05, Student’s *t*‐test. Con, cultured without NK‐92MI cells; +NK, cocultured with NK‐92MI cells. (B) Quantitative real‐time PCR analysis of transcript levels of *CNOT7* and *STAT1* in the four HCC cell lines and L02 cells. The *GAPDH* mRNA level was used for normalization. Transcript levels in L02 cells were used as reference. CNOT7 and STAT1 proteins were determined by western blotting, using GAPDH for normalization. Data were expressed as the mean ± SD of individual groups of cells from three separate experiments. Multiple comparisons were made by the SNK‐*q* method. *P < 0.05, one‐way ANOVA. (C) TGF‐β1 production in the four HCC cell lines and L02 cells was analyzed by ELISA. Transcript levels of *TGF‐β1* were detected by quantitative real‐time PCR. Data were expressed as mean ± SD of individual groups of cells from three separate experiments. Multiple comparisons were made by the SNK‐*q* method. *P < 0.05, one‐way ANOVA. Values with different numbers of asterisks differ significantly (*P* < 0.05).

Cell proliferation of the four HCC cell lines was examined by MTT assay after coculture with NK‐92MI cells. NK cell resistance was the highest in HepG2 cells among the HCC cell lines tested (*P* < 0.05; Fig. 2A). CNOT7 and STAT1 mRNA and protein expression levels in the four HCC cell lines and L02 cells were analyzed by quantitative real‐time PCR and western blotting separately (Fig. 2B). *CNOT7* mRNA levels were remarkably higher in the four HCC cell lines than in L02 cells (*P* < 0.05) and were the highest in HepG2 cells among the HCC cell lines (*P* < 0.05). *STAT1* mRNA expression in HepG2 cells was lower than that in PLC/PRF/5 and SMMC7721 cells (*P* < 0.05). The patterns of CNOT7 and STAT1 protein expression in the four HCC cell lines and L02 cells were similar to those observed for mRNA expression, albeit the differences were less pronounced. CNOT7 and STAT1 protein levels were confirmed by immunohistochemistry results. TGF‐β1 protein and mRNA levels were detected by ELISA and quantitative real‐time PCR in HepG2, SMMC7721, Huh7, PLC/PRF/5 and L02 cells (Fig. 2C). TGF‐β1 protein and mRNA levels were obviously higher in the four HCC cell lines than in L02 cells (*P* < 0.05) and were the highest in HepG2 cells among the HCC cell lines (*P* < 0.05). These data suggested that CNOT7 may be relevant to NK cell resistance in HCC.

### Modulation of CNOT7 expression alters TGF‐β1 secretion in HCC and IFN‐γ secretion in NK cells

**Fig. 3:**
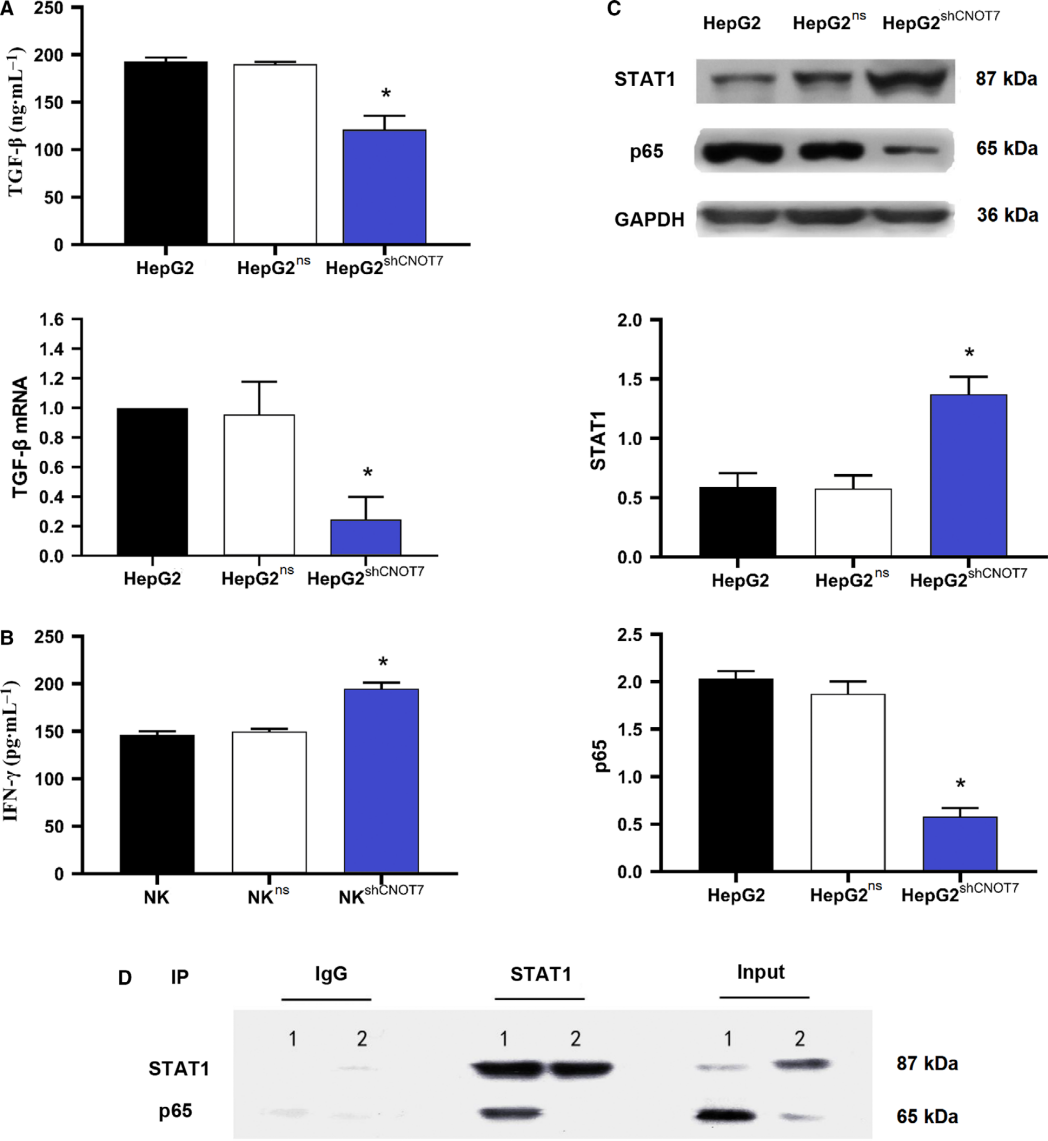
Modulation of CNOT7 expression alters TGF‐β1 secretion in HCC and IFN‐γ production in NK cells. (A) TGF‐β1 production in HepG2, HepG2^ns^ and HepG2^shCNOT7^ cells was analyzed by ELISA. Transcript levels of *TGF‐β1* were detected by quantitative real‐time PCR. Data were expressed as the mean ± SD of individual groups of cells from three separate experiments. Multiple comparisons were made by the SNK‐*q* method. *P < 0.05, one‐way ANOVA. (B) IFN‐γ production by NK‐92MI cells was determined by ELISA. NK^ns^, cultured in HepG2^ns^ cell supernatant; NK^shCNOT7^, cultured in HepG2^shCNOT7^ cell supernatant. Data were expressed as mean ± SD of individual groups of cells from three separate experiments. Multiple comparisons were made by the SNK‐*q* method. *P < 0.05, one‐way ANOVA. (C) STAT1 and NF‐κB p65 protein levels were analyzed by western blotting, using GAPDH for normalization. Data were expressed as mean ± SD of individual groups of cells from three separate experiments. Multiple comparisons were made by the SNK‐*q* method. *P < 0.05, one‐way ANOVA. (D) STAT1 and NF‐κB p65 interaction. Extracts from HepG2 cells transfected with shCNOT7 and nonspecific shRNA were immunoprecipitated with IgG or anti‐STAT1 antibody. Immunoprecipitates were then analyzed by immunoblotting with anti‐p65 antibody. 1: HepG2^ns^; 2: HepG2^shCNOT7^. The experiments were conducted in triplicate.

Considering the earlier results, HepG2 cells were selected as the most suitable cell line for further evaluation of the mechanism of NK cell resistance in HCC. To explore the role of CNOT7 in NK cell resistance of HCC, we detected TGF‐β1 production by CNOT7‐knockdown HepG2 cells and IFN‐γ secretion of NK cells. HepG2 cells were transfected with *CNOT7*‐specific shRNA (HepG2^shCNOT7^) or nonspecific shRNA (HepG2^ns^) as a control. In a previous study, we found that the knockdown effect of shCNOT7 #2 was stronger than that of the other shRNAs tested (*P* < 0.05), and that the STAT1 protein level was higher in HepG2^shCNOT7^ cells than in HepG2^ns^ and HepG2 cells (*P* < 0.05). In this study, ELISA and quantitative real‐time PCR revealed that TGF‐β1 production in HepG2^shCNOT7^ cells was significantly down‐regulated compared with that in HepG2^ns^ and HepG2 cells (Fig. 3A; *P* < 0.05). NK‐92MI cells were cultured in supernatants of HepG2, HepG2^ns^ and HepG2^shCNOT7^ cells for 12 h, and IFN‐γ levels were compared. IFN‐γ secretion of NK‐92MI cells was more obviously up‐regulated in HepG2^shCNOT7^ supernatant than in HepG2^ns^ and HepG2 supernatants (*P* < 0.05; Fig. 3B).

Nuclear factor‐κB (NF‐κB) is an indispensable transcription factor in eukaryotic cells. Activated NF‐κB subunit p65 is translocated to the nucleus, and there promotes *TGF‐β1* transcription [18, 21]. Nuclear p65 protein and *p65* mRNA expression in HepG2, HepG2^ns^ and HepG2^shCNOT7^ cells was determined by quantitative real‐time PCR and western blotting (Fig. 3C). Nuclear p65 protein levels were remarkably decreased in HepG2^shCNOT7^ cells when compared with HepG2^ns^ and HepG2 cells, whereas STAT1 showed the opposite trend (*P* < 0.05). However, there was no significant difference in *p65* mRNA expression between HepG2^shCNOT7^, HepG2^ns^ and HepG2 cells (data not shown). Previous publications indicated the binding of NF‐κB p65 to STAT1 in macrophages [22, 23]. These results caused us to explore a probable physical interaction between NF‐κB p65 and STAT1 proteins in HCC cells. The co‐IP assay revealed a clear interaction between STAT1 and p65 in HepG2 cells (Fig. 3D).

### *CNOT7* knockdown reverses NK cell resistance in HepG2 cells

**Fig. 4:**
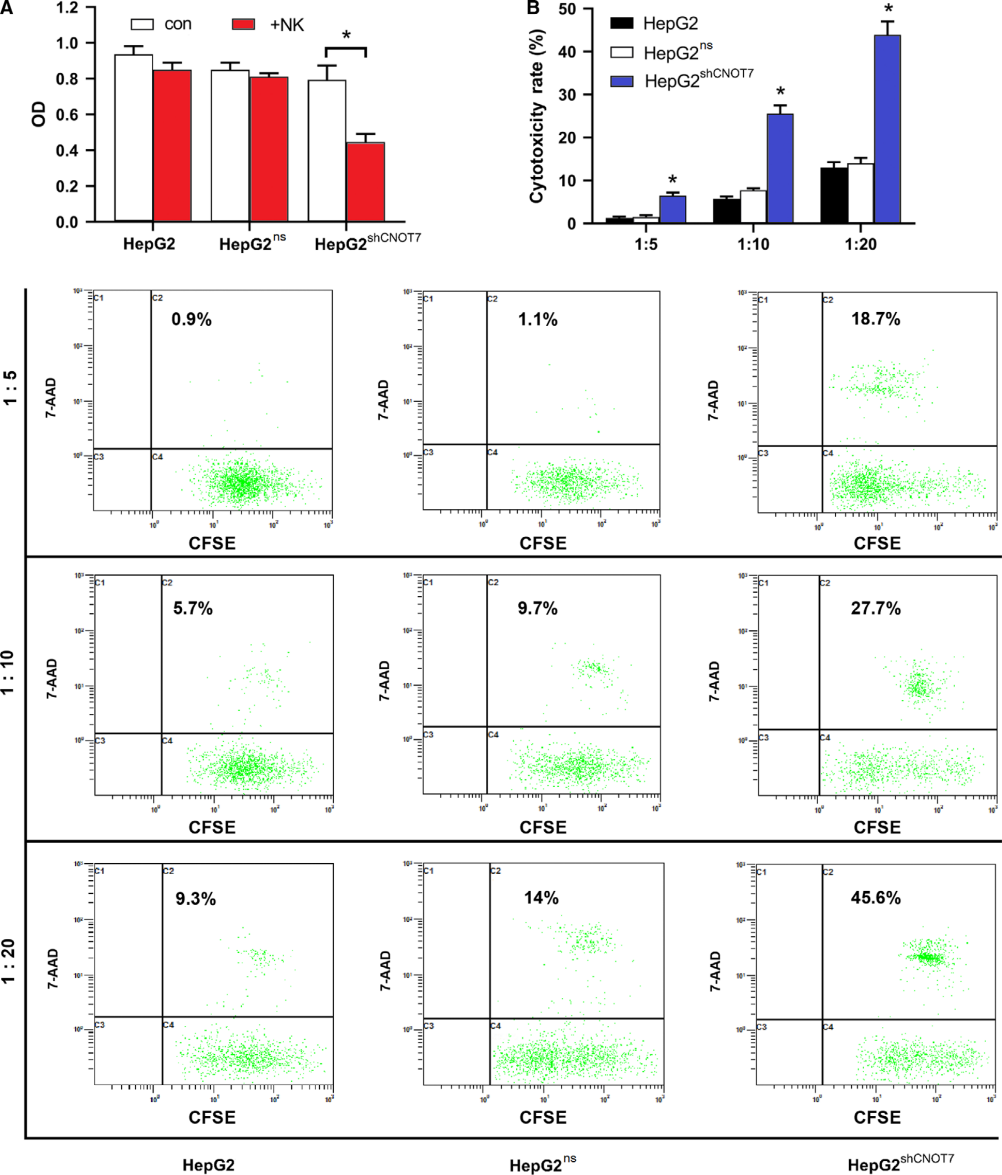
CNOT7 protein depletion reverses NK cell resistance in HepG2 cells. (A) HepG2, HepG2^ns^ and HepG2^shCNOT7^ cells were cocultured with or without NK‐92MI cells at a 1 : 10 ratio for 12 h. MTT assay was applied for cell proliferation detection. Quantitative data (mean ± SD) of individual groups of cells were shown. Experiments were conducted in triplicate; *P < 0.05, Student’s *t*‐test. (B) Cytotoxicity was measured at various E:T ratios by flow cytometry and CFSE/7‐AAD staining. The right upper quadrant represents apoptotic cells. The experiments were performed in triplicate. Data were expressed as mean ± SD of individual groups of cells from three separate experiments. Multiple comparisons were made by the SNK‐*q* method. *P < 0.05, one‐way ANOVA.

To investigate the impact of *CNOT7* knockdown on the sensitivity of HCC cells to NK‐92MI cell cytolysis, we cocultured HepG2^shCNOT7^, HepG2^ns^ and HepG2 cells with NK‐92MI cells at a 1 : 10 E:T ratio for 12 h. NK‐92MI cells remarkably inhibited the proliferation of HepG2^shCNOT7^ cells, but not in HepG2^ns^ and HepG2 cells as indicated by MTT assay (*P* < 0.05; Fig. 4A). Evaluation of cytotoxicity by flow cytometry and CFSE/7‐AAD staining showed the following coculture with NK‐92MI cells at different E:T ratios for 12 h, and the apoptotic rates of HepG2^shCNOT7^ cells increased significantly, suggesting that HepG2^shCNOT7^ cells were obviously more sensitive to NK‐92MI cell cytotoxicity (*P* < 0.05; Fig. 4B). Further, cytotoxicity increased significantly with increasing E:T ratio. These results suggested that CNOT7 knockdown could reverse NK cell resistance in HepG2 cells.

### NK cell surface CD107a expression is up‐regulated, and caspase‐3 expression in CNOT7‐deficient HepG2 cells is significantly enhanced under coculture

**Fig. 5:**
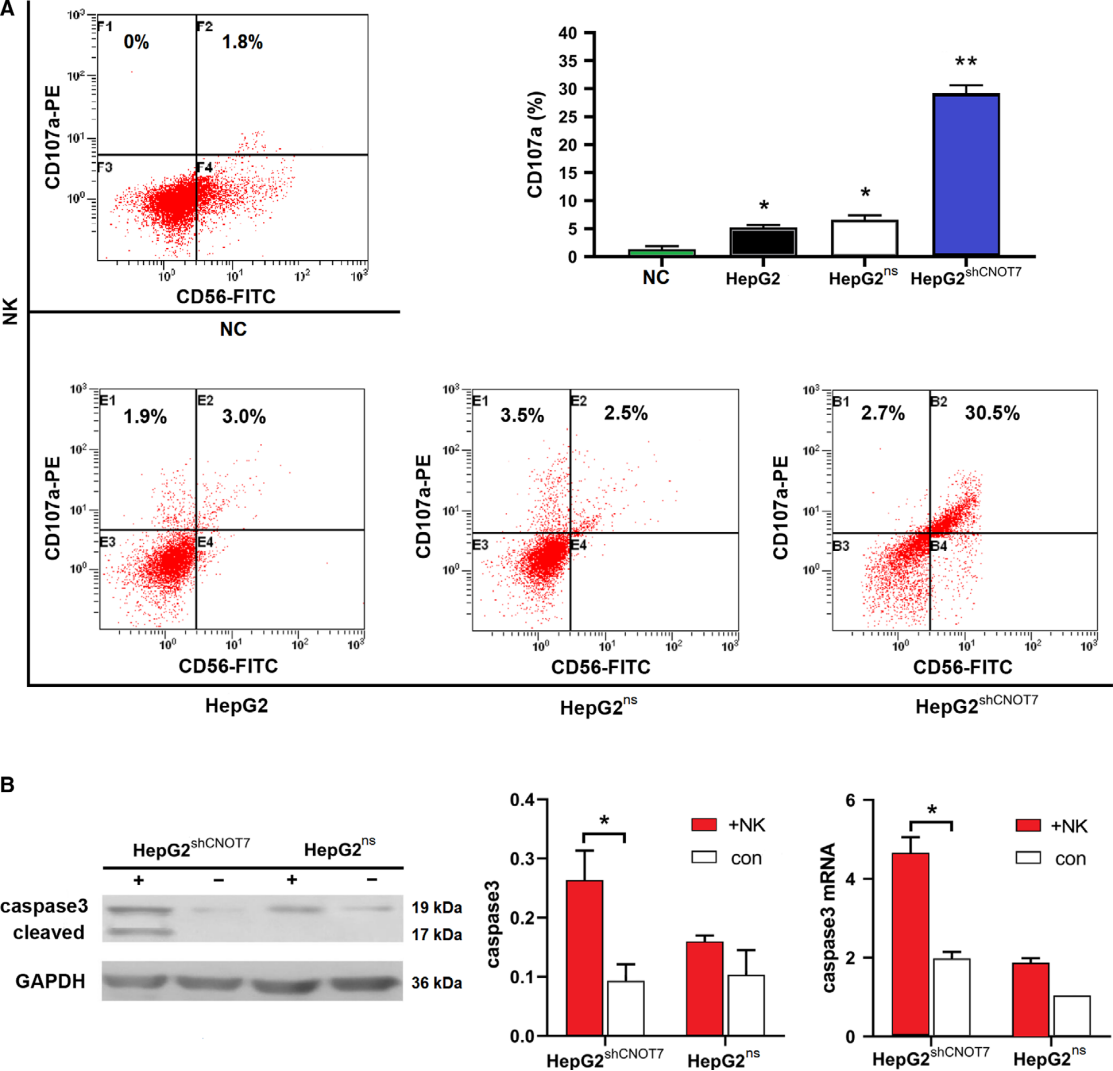
NK cell cytotoxic immune functions are altered upon coculture with CNOT7‐deficient HepG2 cells. (A) To evaluate the cytotoxic immune function, we cocultured NK‐92MI cells with target tumor cells at a 1 : 10 ratio for 3.5 h. CD107a expression of NK‐92MI cells was analyzed by flow cytometry. The upper quadrant indicates that cells showed CD107a expression. Data were expressed as mean ± SD of individual groups of cells from three separate experiments. Multiple comparisons were made by the SNK‐*q* method. *P < 0.05, one‐way ANOVA. (B) HepG2^shCNOT7^ and HepG2^ns^ cells were cocultured with or without NK‐92MI cells at a 1 : 10 ratio for 12 h. Caspase‐3 expression was measured by western blotting and quantitative real‐time PCR. Plus signs (+) indicate cultured with NK‐92MI cells; minus signs (–) indicate cultured without NK‐92MI cells. Quantitative data (mean ± SD) of individual groups of cells were shown. Experiments were conducted in triplicate; *P < 0.05, Student’s *t*‐test. Values with different numbers of asterisks differ significantly (*P* < 0.05)

To evaluate the cytotoxic immune function, we cocultured target tumor cells with NK‐92MI cells at a 1 : 10 ratio for 3.5 h and an NC. Surface CD107a expression on NK‐92MI cells was detected by flow cytometry. Although CD107a expression was higher in HepG2 and HepG2^ns^ cells than in the NC (*P* < 0.05), CD107a expression was obviously enhanced in coculture with HepG2^shCNOT7^ (*P* < 0.05), but not in HepG2 and HepG2^ns^ cells (Fig. 5A). To elucidate the mechanism underlying cytotoxicity toward target tumor cells, we cocultured target tumor cells with NK‐92MI at a 1 : 10 ratio for 12 h, and caspase‐3 expression was determined by quantitative real‐time PCR and western blot. Caspase‐3 expression was remarkably enhanced in HepG2^shCNOT7^ (*P* < 0.05), but not in HepG2 and HepG2^ns^ cells. Changes in *CASPASE‐3* mRNA expression showed similar patterns (Fig. 5B).

## Discussion

HCC is one of the most common malignant tumors worldwide [1]. HCC treatment is challenged not only by the tumor immune depression microenvironment blocking antineoplastic activity in the host, such as in many other cancers, but also because HCC is located in the liver, which is an immunologically privileged organ [17]. NK cells are gathered in the liver and represent up to 30–50% of total intrahepatic lymphocytes [6]. NK cells play an important role in the antitumor immune reaction, because they can regulate tumor proliferation and metastasization [9]. However, HCC cells can escape from NK cell‐mediated immunosurveillance within the permissive tumor microenvironment [4, 5]. Targeting the tumor microenvironment could be an effective solution for eliminating cancer and reducing the risk for recurrence.

CNOT7 plays a vital factor in regulating major histocompatibility complex I mRNA deadenylation [24]. In MCF7 cells, CNOT7 knockdown resulted in STAT1 overexpression and a hyperactivated subset of STAT1‐regulated genes, leading to stopping or slowing of cell growth [16, 25, 26]. In this study, higher CNOT7 expression was associated with the poor prognosis of patients with HCC. CNOT7 expression was increased, whereas STAT1 expression was decreased in HCC tumor tissues. TGF‐β1 is a powerful immune suppressor secreted from cancer cells of many cancer types, including HCC [11, 12, 13, 27]. Plasma concentration of TGF‐β1 was positively correlated with the degree of tumor progression in patients with lung or colorectal cancer [28]. TGF‐β1 inhibits cytokine secretion and cytotoxicity of NK cells [15, 16, 17, 18, 27]. NK cell function was impaired, which had been widely observed in patients with advanced cancer [29, 30, 31]. Consistent with previous data, we found that plasma TGF‐β1 levels were obviously elevated in patients with HCCBC, albeit only slightly higher in adjacent tumor tissues than in tumors and healthy tissues. One possible explanation is that abundant CD4^+^ CD25^+^ regulatory T cells were activated by tumor‐derived TGF‐β1, which creates a robust inhibitory network in solid tumors [32]. IFN‐γ is a cytokine that is critical in both innate and acquired immunity in humans. It has been suggested to be the main factor involved in the immune surveillance of cancer cells [33]. However, the role of IFN‐γ in carcinogenesis is complex. IFN‐γ signaling inhibits HCC tumor growth in some studies but has also been reported to be associated with the resistance of HCC to therapy [34, 35]. Our previous study demonstrated that IFN‐γ resistance correlated with the level of CNOT7 overexpression and STAT1 deficiency. CNOT7 is a key factor in IFN‐negative regulation [16, 25]. In this experiment, we found that IFN‐γ concentration was up‐regulated only in the plasma of patients with HCCBC and HBC, whereas IFN‐γ was obviously higher in normal liver tissues than in adjacent and tumor tissues. One of the reasons may be that tumor‐derived TGF‐β1 created a strong inhibitory tumor network and impaired immune cells, such as NK cells, in cytokine secretion functions [11, 12, 13].

We found that TGF‐β1 mRNA and protein levels were significantly higher in four HCC cell lines than in L02 cells. CNOT7 and TGF‐β1 were positively correlated with NK cell resistance, whereas STAT1 was negatively correlated. Furthermore, *CNOT7* knockdown (HepG2^shCNOT7^) significantly promoted STAT1 protein expression and inhibited cytokine TGF‐β1 secretion. NF‐κB is a family of dimeric transcription factors that are found in every eukaryotic cell. RelA (p65) is a distinct protein of the NF‐κB family. In resting cells, most NF‐κB proteins stay in the cytoplasm [35]. In HCC models, the NF‐κB pathway was found to associate with cancer progression [35, 36]. Activated NF‐κB p65 forms a dimer that can be translocated to the nucleus, where it promotes *TGF‐β1* transcription [18, 21]. Here, through co‐IP analysis, we demonstrated a direct interaction between STAT1 and p65 proteins. STAT1/p65 dimer cannot translocate to the nucleus [22, 23]. Nuclear p65 protein levels were remarkably decreased, inhibiting *TGF‐β1* transcription. Therefore, our results indicate that CNOT7 may be a positive regulator of TGF‐β1 secretion by controlling p65 trafficking. High concentration of TGF‐β1 secreted by HCC is closely related to immune cell depletion [11, 12, 13]. Recent studies have reported that TGF‐β1 adequately impaired ATP‐coupled respiration in CD4^+^ T cells and specifically inhibited ATP synthase activity and inhibited IFN‐γ secretion by CD4^+^ T cells [37]. TGF‐β1/Smad3 suppressed IFN‐γ by transcriptionally suppressing E4BP4, a master transcription factor in NK cell development [38]. We cultured NK cells in supernatants of HepG2^shCNOT7^, HepG2^ns^ and HepG2 cells, and compared IFN‐γ levels. Results show that NK cells secreted obviously higher amounts of IFN‐γ in HepG2^shCNOT7^ supernatant than in HepG2 supernatant.

We previously demonstrated that CNOT7 could inhibit cytoplasmic STAT1 trafficking and block IFN‐γ/STAT1 signaling, and that *CNOT7* knockdown reversed IFN‐γ resistance in HCC cells [16]. In this experiment, our results indicated that *CNOT7* knockdown altered TGF‐β1 production of HCC and IFN‐γ production of NK cells, suggesting that *CNOT7* knockdown might reverse NK cell resistance by improving the tumor immune microenvironment of HCC. To validate this assumption, we cocultured NK cells with HepG2^shCNOT7^ and HepG2 cells at various E:T ratios. The proliferation of HepG2^shCNOT7^ cells was obviously inhibited by NK cells, and cytotoxicity of NK cells was markedly up‐regulated. Because CD107a serves as a functional marker of NK cell activity [39], we analyzed changes in CD107a expression on NK cells cocultured with target tumor cells. CD107a expression was significantly enhanced in cocultured with HepG2^shCNOT7^ cells, indicating that numerous NK cells were activated. In experiments conducted to elucidate the mechanisms underlying the cytotoxicity toward target tumor cells, we found that caspase‐3 expression and cleavage were significantly enhanced in HepG2^shCNOT7^ cells after coculture. Cleaved caspase‐3 is viewed as a reliable marker of dead or apoptotic cells [40, 41]. *CNOT7* knockdown did not affect HCC cell proliferation in our studies. This suggests that this method has fewer side effects than other methods.

In summary, our results demonstrate that NK cell resistance in HCC correlated with CNOT7 overexpression levels. Given that modulation of CNOT7 expression altered TGF‐β1 secretion in HCC and IFN‐γ secretion by NK cells, and that CNOT7 is a pivotal factor in IFN‐negative regulation, CNOT7 depletion appears to be highly effective in the inhibition of HCC cell proliferation *in vitro*. CNOT7 improves tumor‐induced immune tolerance, opening potential new routes to the development of new therapeutic approaches against liver cancer. Furthermore, given the observed immune‐enhancing effect, CNOT7 depletion could represent an effective adjuvant therapy in immunotherapy for HCC to prevent microbial infections and inflammation‐associated disease.

## Conflict of interest

The authors declare no conflict of interest.

## Author contributions

CR and XR wrote the manuscript. CR, XR, DC and Haichao Zhao performed the majority of experiments. ZZ and HL did the analysis. YL and XF drew figures. Haoliang Zhao and JH designed the study and revised the manuscript.
